# *Porphyromonas gingivalis*: An Overview of Periodontopathic Pathogen below the Gum Line

**DOI:** 10.3389/fmicb.2016.00053

**Published:** 2016-02-09

**Authors:** Kah Yan How, Keang Peng Song, Kok Gan Chan

**Affiliations:** ^1^Division of Genetics and Molecular Biology, Institute of Biological Sciences, Faculty of Science, University of MalayaKuala Lumpur, Malaysia; ^2^School of Science, Monash University Sunway CampusSubang Jaya, Malaysia

**Keywords:** *Porphyromonas gingivalis*, oral pathogen, periodontal disease, gingivitis, virulence factors, inflammatory response

## Abstract

Periodontal disease represents a group of oral inflammatory infections initiated by oral pathogens which exist as a complex biofilms on the tooth surface and cause destruction to tooth supporting tissues. The severity of this disease ranges from mild and reversible inflammation of the gingiva (gingivitis) to chronic destruction of connective tissues, the formation of periodontal pocket and ultimately result in loss of teeth. While human subgingival plaque harbors more than 500 bacterial species, considerable research has shown that *Porphyromonas gingivalis*, a Gram-negative anaerobic bacterium, is the major etiologic agent which contributes to chronic periodontitis. This black-pigmented bacterium produces a myriad of virulence factors that cause destruction to periodontal tissues either directly or indirectly by modulating the host inflammatory response. Here, this review provides an overview of *P. gingivalis* and how its virulence factors contribute to the pathogenesis with other microbiome consortium in oral cavity.

## Introduction

Periodontal diseases are complex, multifactorial, polymicrobial infections characterized by the destruction of tooth-supporting tissues. The disease begins as acute inflammation of the gingival tissue and untreated infections can progress to formation of teeth pockets, and eventually loss of teeth. According to the World Health Organization, periodontal disease affects 10–15% of adult populations worldwide (Petersen and Ogawa, 2012).

Substantial data accumulated over the years has implicated the involvement of only a small proportion of bacteria, which reside in the subgingival niche, in the initiation and progression of periodontal disease. There is strong evidence that points to *Porphyromonas gingivalis*, a Gram-negative anaerobes, as the keystone species in the development of chronic periodontitis.

The past decades of extensive research on *P. gingivalis* have produced various lines of evidences on the contribution of this anaerobe to the progression of periondontal disease. These findings are important in increasing our understanding on the virulence characteristics and cellular interaction between *P. gingivalis* and the host, thereby elucidating potential therapeutic approaches to control periodontal disease progression.

## The Oral Flora and Normal Periodontium

The oral cavity possesses a number of features which make it a distinct habitat for a menagerie of microorganisms. The surfaces in the oral cavity are continuously bathed in saliva most of the time at a narrow temperature range (34 to 36°C) and a pH close to neutrality (Marcotte and Lavoie, 1998). With such an ideal environment, various classes of microflora are found to be distributed in various ecological niches (Parahitiyawa et al., 2010).

In general, the mouth harbors at least six billion bacteria which are represented by more than 700 species (Theilade, 1990; Aas et al., 2005), as well as other types of microorganisms, including fungi, mycoplasma, protozoa, and possibly even viruses (Pennisi, 2005). Generally, oral bacteria can be broadly classified as Gram-positive and Gram-negative bacteria, and secondarily as either anaerobic or facultatively anaerobic according to their oxygen requirements. Some of the more frequently isolated microorganisms in human oral cavity are listed in **Table 1**.

**Table 1:** The predominant human oral microbiota.

Microbial group	Microbial genus/species
**Gram-positive**	
Aerobic or facultative	*Streptococcus (S. gordonii, S. mitis, S. auralis, S. salivarius)*
	*Staphylococcus (S. aureus, S. epidermidis)*
	*Enterococcus (E. faaecalis)*
	*Lactobacillus (L. casei, L. fermentum)*
	*Corynebacterium (C. matruchotii)*
	*Actinomyces (A. naeslundii, A. israelli, A. viscosus)*
	*Arachnia (A. propionica)*
	*Rothia (R. dentocariosa)*
Obligate anaerobes	*Bacillus (B. cereus)*
	*Propionibacterium (P. acnes)*
	*Peptostreptococcus (P. micros, P. anaerobius)*
**Gram-negative**	
Aerobic or facultative	*Campylobacter (C. rectus, C. concisus, C. gracilis)*
	*Actinobacillus (A. actinomycetemcomitans)*
Obligate anaerobes	*Fusobacterium (F. nucleatum)*
	*Porphyromonas (P. gingivalis)*
	*Prevotella (P. melaninogenica, P. oralis, P. intermedia)*

Despite the diverse community of oral microbiota, the oral cavity is, nonetheless, characterized by a stable community known as the climax community. Therefore, if imbalance in the oral resident microbiota occurs, oral diseases such as caries and periodontal diseases seem to appear, leading to multiplication of potentially pathogenic microorganisms. Several studies have illustrated that a change in microbial species in the gingival sulcus from Gram-positive, facultative, fermentative microorganisms to predominantly Gram-negative, anaerobic, chemoorganotrophic, and proteolytic organisms has been highly associated with destruction of periodontal tissue (Eloe-Fadrosh and Rasko, 2013).

## Periodontal Diseases

Periodontal disease generally refers to inflammatory pathologic state of the gingiva and the supporting structures of the periodontium which include gingival, alveolar bone, periodontal ligament, and cementum. They are commonly found in most human populations and result in significant morbidity, with exfoliation of the teeth in severe condition. In United States, recent epidemiological data suggests that periondontal disease affects one-half of its population over 30 years of age and is the major cause of tooth loss among adults (Bostanci and Belibasakis, 2012). According to the periodontal disease classification system proposed by the American Academy of Periodontology (AAP), periodontal diseases are generally grouped into two major categories, gingival diseases, and periodontitis, depending on whether destruction of the periodontal attachment has occurred (Wiebe and Putnins, 2000).

Gingival disease is defined as inflammation of the gingival tissues caused by accumulation of dental plaque and is characterized clinically by redness, swelling, and bleeding of the tissues. As the periodontal ligament and alveolar bone are not involved in this event, the attachment of the teeth is not affected (Williams et al., 1992). Gingivitis can remain indefinitely for a long period and will not progress to periodontitis, unless there are perturbations in local conditions or generalized host susceptibility (Offenbacher, 1996).

On the other hand, periodontitis refers to the irreversible plaque-induced inflammation of the periodontal tissues leading to destruction of the periodontal ligament and alveolar bone, and migration of the epithelial ligament. Subsequently, this causes formation of a periodontal pocket, the main clinical feature of periodontitis (Williams et al., 1992). This pocket is an ideal surface for bacterial colonization and the formation of subgingival plaque.

Various studies have shown that periodontitis occurs more often among patients with systemic diseases such as diabetes mellitus, AIDS, leukemia, and Down’s syndrome (Komatsu, 2014; Mehta, 2015). A growing body of evidence suggests that periodontitis may enhance the risk for several potentially deadly conditions including cardiovascular diseases (e.g., heart attack, coronary artery disease, and stroke) and diabetes (Saremi et al., 2005; Friedewald et al., 2009). According to a report by AAP in 1996, it was found that people with some forms of gum disease are almost twice as likely to suffer from coronary artery disease compared to those with healthy gum.

**FIGURE 1:**
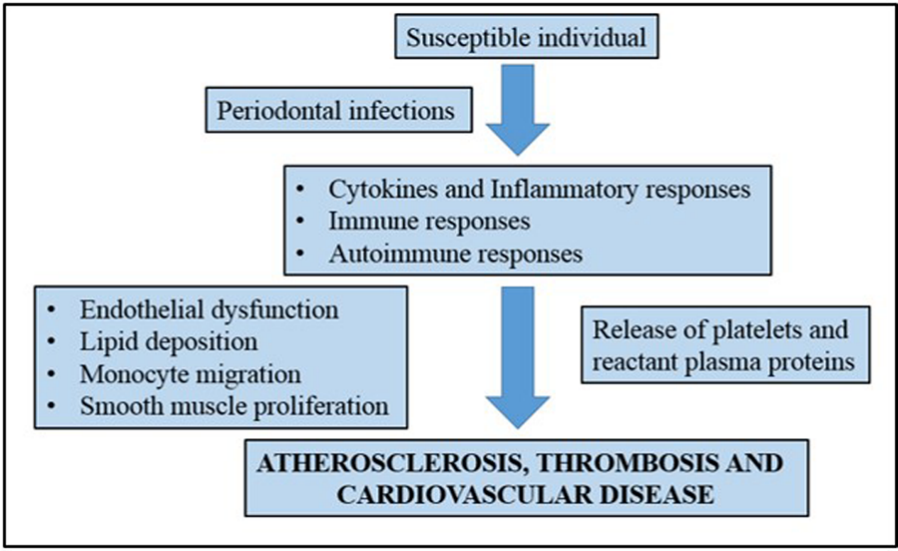
**A simplified representation showing the association of chronic periodontal inflammation and pathogenesis of cardiovascular disease.** Adapted from Bartold and Narayanan (2006).

For many years, numerous studies have been developed to show the possible connection between periodontal disease and cardiovascular disease. Recent studies, such as the study of Nakano et al. (2006) attempted to show the direct mechanisms that link periodontal diseases to cardiovascular disease. A number of mechanisms have been proposed to explain this association including a common factor that predisposes certain individuals to hyper-inflammatory response in cardiovascular disease (Beck et al., 1996). It is believed that oral pathogens could enter the bloodstream and the inflammation caused by periodontal disease increases plaque build up, which subsequently contributes to dilation of the arteries (Bartold and Narayanan, 2006). **Figure 1** shows a simplified diagram of the potential roles that periodontal inflammation might play in the pathogenesis of cardiovascular diseases.

*Streptococcus mutans*, a major pathogenic agent of dental caries, was detected in unusual high frequency in both heart valve tissues and atheromatous plaque samples than any other species (Nakano et al., 2006). In fact, together with *P. gingivalis, S. mutans* was previously found to accelerate atherogenic plaque formation in a murine model and induce platelet aggregation, which presumably leads to thrombus formation (Herzberg and Meyer, 1996; Kuramitsu et al., 2001). The latest study by Oliveira et al. (2015) showed that *S. mutans, Prevotella intermedia, P. gingivalis*, and *Treponema denticola* were frequently detected in cardiac valve samples using polymerase chain reaction (PCR) method, corroborating previous findings. The high detection rate of *S. mutans* in dental plaque samples may suggest that the *S. mutans* found in the valve samples was originated from the oral cavity. However, there is a concern of the possibility of detecting DNA of dead bacteria due to the high sensitivity of real-time PCR.

On the other hand, the American Heart Association noted that neither has periodontal disease been proven to cause atherosclerotic vascular disease nor has the treatment of periodontal disease been proven to prevent atherosclerotic vascular disease (Lockhart et al., 2012). This was supported by a report from Hayashi et al. (2015) in which there is an absence of strong correlation between periodontal condition and parameters of arteriosclerosis status in patients with coronary artery disease. This study, however, also has some limitations such as patients without coronary artery disease were excluded and biomarker samples were not collected from different regions in oral cavity to reflect local imflammatory conditions.

It was reported that periodontal disease and cardiovascular disease share common risk factors such as smoking, diabetes mellitus, aging, and obesity (Genco and Borgnakke, 2013). Systemic inflammatory markers were also detected from both diseases. Hence, evidence regarding a correlation between these two diseases remains a challenging study. Other common factors including lifestyle and personal hygiene also played important roles in both diseases. With much conflicting literature, there is a need to have additional long-term studies to provide a strong causative link between periodontal and heart diseases.

Besides cardiovascular disease, *in vitro* and animal model studies suggest that *P. gingivalis* can breach immune tolerance in susceptible individuals and exacerbate rheumatoid arthritis through enzymatic modification of host proteins (Maresz et al., 2013). Apart from that, periodontal disease has also been found to be a risk factor for pre-term low birth weight babies (Ali and Abidin, 2012). The inflammatory mediators that occur in periodontal disease have critical roles in the initiation of labor and there are plausible mechanisms that could link the two conditions (Williams et al., 2000). Based on epidemiological and microbiological immunological studies, the postulated mechanisms include translocation of periodontal pathogens to the fetoplacental unit and action of a periodontal reservoir of virulence factors as inflammatory mediators. However, the relationship between adverse pregnancy outcomes and maternal antibody response to *P. gingivalis* awaits further investigation (Madianos et al., 2013).

## Bacterial Etiology in Periodontal Diseases

Major advances have been made in these few decades in understanding the pathogenesis and natural history of periodontal diseases. Nevertheless, studies conducted in the 1930s to 1970s were unable to identify specific bacteria as etiological agents of periodontal diseases. As such, the “non-specific theory” was suggested, which hypothesizes that periodontal disease is due to a consortium of microorganisms rather than the importance of any bacterial species as the sole causative agents (Theilade, 1986). However, in the late 1970s and after, more specific microorganisms were isolated as etiological agents of periodontitis (Tanner et al., 1979; Slots et al., 1986; Moore and Moore, 1994).

It is now widely accepted that a myriad of bacteria and not a single microorganism, are involved in periodontal diseases. In fact, the onset of periodontal tissue inflammation is triggered by the colonization of the subgingival region by periodontal bacteria. On the tooth surfaces, for example, early or primary colonizers are mainly streptococci and actinomyces. Over time, the proportions of these Gram-positive facultatively anaerobic bacteria decrease and eventually Gram-negative anaerobes become more established, especially at the interface of the teeth and gums (Jenkinson and Lamont, 2006).

Nonetheless, complex interactions between bacterial flora and the host defense mechanisms significantly influence the balance between bacterial aggression and host protection and thus determines whether periodontal breakdown occurs (Hajishengallis et al., 2012). In light of these criteria, a number of experimental evidences have demonstrated that the primary etiological agents of periodontal diseases are generally Gram-negative rods which include *Actinobacillus actinomycetemcomitans, Tannerella forsythia* (previously designated *Bacteroides forsythus*), *Prevotella, Fusobacterium*, and *P. gingivalis.* Not one of these microbial species is capable of causing the destructive events involved in the periodontal disease progression but the etiology requires a concerted interaction of these members to establish their niches in the oral cavity (Marcotte and Lavoie, 1998; Maiden et al., 2003; Paster et al., 2006).

## *Porphyromonas Gingivalis* and its Prevalence

**FIGURE 2:**
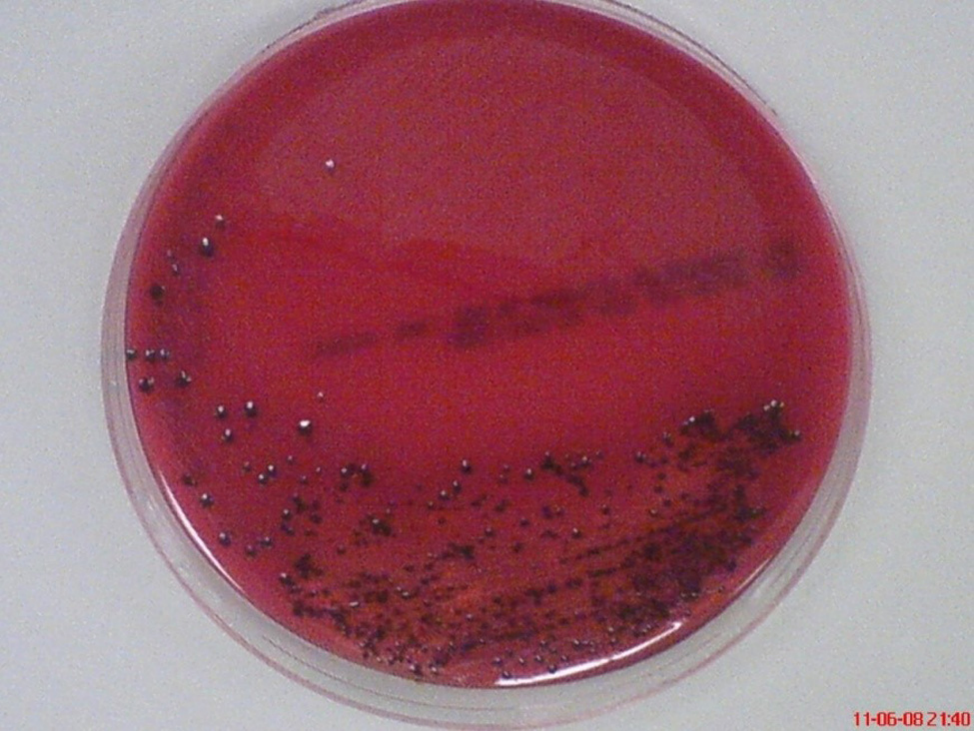
**Black-pigmented colonies of periodontopathogen *P. gingivalis* on horse blood agar.** The pigment is thought to be related to accumulation of hemin (oxidized form of heme) on the cell surface when grown on blood agar.

Among major periodontal pathogens, *P. gingivalis* appears to be one of the prime etiological agents in the pathogenesis and progression of the inflammatory events of periodontal disease (Hajishengallis et al., 2012). This periodontopathic bacterium was found in 85.75% of subgingival plaque samples from patients with chronic periodontitis (Datta et al., 2008). This non-motile, asaccharolytic, Gram-negative bacterium is an obligately anaerobic rod which forms black-pigmented colonies on blood agar plates (**Figure 2**). It has an absolute requirement for iron in its growth. It was formerly named *Bacteroides gingivalis* prior to its reclassification as a new genus, *Porphyromonas* (Nisengard and Newman, 1994). The name *Porphyromonas* comes from the Greek adjective *porphyreos* meaning purple and the Greek noun *monas* meaning unit. Hence, the word *Porphyromonas* means porphyrin cell as the colonies on blood agar plates turn black after 6 to 10 days due to heme accumulation (Shah and Collins, 1988).

The major habitat of *P. gingivalis* is the subgingival sulcus of the human oral cavity. It relies on the fermentation of amino acids for energy production, a property required for its survival in deep periodontal pocket, where sugar availability is low (Bostanci and Belibasakis, 2012). Being an obligate anaerobe, *P. gingivalis* serves as the secondary colonizer of dental plaque, often adhering to primary colonizers such as *Streptococcus gordonii* and *P. intermedia.* A study by Bodet et al. (2006) demonstrated that this asaccharolytic bacterium is associated with *T. denticola* and *T. forsythia* to form the red bacterial complex which is highly recognized in advanced periodontal lesions. Additional evidence for the presence of *P. gingivalis* has also came from immunological studies (Moore et al., 1991; Schmidt et al., 2014). Virtually, all investigators agree that serum antibody levels to *P. gingivalis* are higher in patients diagnosed with adult periodontitis (Mahanonda et al., 1991; Casarin et al., 2010).

In the past few decades, *P. gingivalis* strains have been classified into invasive and non-invasive strains based on their ability to form abscesses in a mouse model. It has been demonstrated that the invasive strain of *P. gingivalis* possesses more pathogenic activities than the non-invasive strain both *in vitro* and *in vivo* (Dorn et al., 2000; Baek et al., 2015).

The presence of *P. gingivalis* acting either alone or as a mixed infection with other oral pathogens and possibly the deficiency of certain immunological factors in the host appears to be essential for the etiology of advanced periodontitis (Haffajee and Socransky, 1994). The number of *P. gingivalis* has been shown to increase substantially in sites with periodontitis and lower or non-detectable in sites with subgingival health or plaque-associated gingivitis (Schmidt et al., 2014). It usually resides in higher proportion in deep than in shallow periodontal pockets (Ali et al., 1996). About 40–100% of adult periodontitis patients have been infected with these opportunistic bacteria.

With high frequency of *P. gingivalis* in adult periodontitis lesions, it is strongly hypothesized that *P. gingivalis* interact with other members of the host microbiota by synthesizing various pathogenic factors, leading to the progression of the disease. However, how *P. gingivalis* communicates with selective host cells to produce destructive biological molecules and triggers the conversion of a healthy oral tissue to a diseased state is yet to be known (Holt et al., 1999).

## Virulence Factors of *P. gingivalis*

The induction and progression of periodontal tissue destruction are complex processes involving plaque accumulation, release of bacterial substances, and host inflammatory response. *P. gingivalis* is known to produce a repertoire of virulence factors that could penetrate the gingivae and cause tissue destruction directly or indirectly, by induction of inflammation (Hajishengallis et al., 2012). Virulence factors may be defined as the constituents or metabolites of an organism which are essential in various stages of the life cycle and cause damage to the host. The capacity of an organism colonize and evade anti-bacterial host defense mechanisms, as well as the ability to produce substances that could initiate tissue destruction, are integral features of a successful pathogen. Some of the virulence factors are given in **Table 2**.

**Table 2:** The virulence factors and host effectors produced by *P. gingivalis.*

Virulence factors	Effect on host evasion
Enzymes (hyaluronidase, chondroitin sulfatase), capsule	Decrease phagocytosis for invasion, chemotaxis inhibitors
Lipopolysaccharide	Bone resorption, Immunoglobulin proteases
Fimbriae, exopolysaccharide, outer membrane proteins	Adhesion or attachment to host outer membrane
Collagenase, trypsin-like protease, gelatinase	Degradation of plasma protease inhibitors, destruction of periodontal tissue
Aminopeptidase	Degradation of iron transport protein

In order to survive and multiply in a host, the invading pathogen needs to overcome the host external protective barriers before it could find a suitable ecological niche for colonization. Colonization of the host tissues could only happen in the presence of virulence factors such as fimbriae, capsules, lipopolysaccharide (LPS), lipoteichoic acids, haemagglutinins, gingipains, outer membrane proteins, and outer membrane vesicles (Holt et al., 1999; Hajishengallis and Lamont, 2014). The expression of virulence factors is often regulated in response to changes in the external environment of the periodontopathogen. If active in a susceptible host, these virulence factors can result in a rapid and significant destruction of periodontal tissues, bone resorption, induction of host responses by cytokine production, as well as inhibition of host protective mechanisms. However, the expression of these virulence determinants and their mechanism during various stages of periodontal disease have not been extensively investigated. To fully understand the specific function of each factor and its mechanism in pathogenesis requires inactivation of the factors coupled with biochemical evaluation and *in vivo* virulence testing.

## Capsules

To get established in the oral cavity, microorganisms must first adhere to teeth or to mucosal surfaces (Yoshimura et al., 2009). Adherence is essential for providing resistance to the flow of saliva. Adherence is usually mediated by adhesins on the surface of bacteria and by receptors on the oral surface. Microbial adhesins are found as cell wall components or are associated with cell structures, such as capsules or fimbriae (Marcotte and Lavoie, 1998). The chemical composition of the capsule differs between strains, mainly in their sugar composition. For instance, *P. gingivalis* ATCC 53977 does not contain galactose but is rich in amino sugars. *P. gingivalis* is found to display at least six serotypes of capsular antigens, which comprises K1–K6 (Laine et al., 1997).

One of the earliest studies reported that highly encapsulated *P. gingivalis* strains exhibit decreased autoagglutination, lower densities and are more hydrophilic than the non-encapsulated strains (Van Steenbergen et al., 1987). According to Singh et al. (2011), increased encapsulation is also correlated with increased resistance to phagocytosis, serum resistance, and decreased induction of polymorphonuclear leukocyte chemiluminescence. In addition, the capsule is also found to be involved in the perturbation of gingival epithelial cells. Studies by Dierickx et al. (2003) revealed that the presence and type of capsule had a significant influence on the initial adhesion of *P. gingivalis* to human periodontal pocket epithelial cells. However, it should be noted that the capsule may possibly interact with surface protein to facilitate attachment to the host cells. On the other hand, the level and mechanism of co-aggregation between *P. gingivalis* and another periodotopathogen, *Fusobacterium nucleatum* has been shown to be capsular dependent (Rosen and Sela, 2006) as well. Another study by Katz et al. (1996) revealed that the virulent *P. gingivalis* W83 and W50 which have thicker capsules cause a decrease in the production of leukocytes than those strains which are less virulent such as strain 376. This clearly indicates that the observed difference in virulence strains is likely due to difference in capsular structure and adhesion capacity.

Studies using mouse infection models have revealed that by shielding microbial surface components, encapsulated *P. gingivalis* strains are more virulent than non-encapsulated strains. Non-encapsulated strains mostly cause non-invasive, localized abscesses whereas encapsulated strains cause invasive, spreading phlegmonous infections after subcutaneous inoculation of experimental animals. The non-encapsulated strains are also subjected to increased phagocytosis or are killed quickly by macrophages and dentritic cells (Laine et al., 1997). On the contrary, in terms of invasion efficiency, the capsule of *P. gingivalis* makes it less efficient in invading gingival fibroblast compared to the non-capsular strains (Irshad et al., 2012). Recent findings by Brunner et al. (2010) demonstrated that encapsulated *P. gingivalis* was able to modulate the host response to bacteria by reducing the synthesis of cytokines interleukin-1 (IL-1), IL-6, and IL-8 by fibroblasts. On top of its immunomodulating properties, capsule was shown to promote virulence using mouse abscess model by reducing phagocytosis and thereby increasing bacterial survival within host cells, and ultimately a long-term inflammatory response (Singh et al., 2011). However, the effect of oxidative stress on expression of capsule was not taken into account as the experiments were performed under anaerobic conditions. Further validations are needed to show a more precise contribution of capsule in pathogenicity. In additional to bacterial survival, capsule may also contribute to increased survival by reducing the bactericidal effect of small antimicrobial peptide known as defensins (Igboin et al., 2011). This study used *Drosophila melanogaster* killing model to characterize the host response to *P. gingivalis* infection. Hence, the findings might not be very significant as *Drosophila* is not a natural host for *P. gingivalis* and host–pathogen interactions could not be studied using this organism.

In the past decade, the regulatory mechanisms in synthesis of capsular surface has been the object of great attention. It is believed that the mechanisms are quite complicated, involving posttranscriptional regulation and expression of biosynthetic machinery from multiple loci. To date, only two different regulatory mechanisms have been identified in *P. gingivalis.* One is a tyrosine phosphatase (Ltp1) encoded by PG1641, which controls expression of a number of genes encoding proteins involved in the synthesis of surface polysaccharides. In the *ltp1* deletion mutant, the expression of K-antigen capsule was downregulated (Maeda et al., 2008). The second regulatory mechanism that has been identified is the DNABII protein HU β-subunit (PG0121) which likewise, caused a decrease in expression of K-antigen synthesis locus in PG0121 mutant (Priyadarshini et al., 2013; Tjokro et al., 2014). The latest study by Bainbridge et al. (2015) demonstrated that an antisense RNA molecule which is encoded within the 77 bp inverted repeat element near the 5′ end of the K-antigen capsule synthesis genes affect the expression of capsular surface in virulent *P. gingivalis* strain W83. Deletion of this element diminished the capsule synthesis and altered the structure of LPS (Bainbridge et al., 2015). This major findings are important in elucidating the regulatory role of secondary structure of RNA in modulating the synthesis of K-antigen capsule. However, it is not known if the antisense RNA is a genuine RNA or encodes for a signaling peptide. Furthermore, it is not known if other regulatory genes may play a role in interacting with other proteins or enzymes in capsule production.

## Fimbriae

Fimbriae are thin, proteinaceous surface appendages that protrude from the outer membrane of a bacterial cell. These 3–25 μm long structures are harbored by most of the *P. gingivalis* strains. Research on *P. gingivalis* fimbriae has a long history going back to the 1980s (Handley and Tipler, 1986). Studies showed that *P. gingivalis* expresses two distinct fimbriae on its cell surface: one consists of a subunit protein (named FimA or fimbrillin) encoded by the *fimA* gene (termed long, or major fimbriae), while the other subunit Mfa protein is encoded by the *mfa1* gene (termed short, minor or Mfa1 fimbriae). Even though the two fimbriae are antigenically distinct and differ in their amino acid composition, they are believed to contribute to the progression of periodontal inflammatory reactions (Amano et al., 2010). Interestingly, a major fimbria-like structure was found in a *P. gingivalis* strain in which neither FimA nor Mfa1 fimbriae was detected. This 53 kDa novel fimbriae is another major outer membrane protein of *P. gingivalis* (Nagano et al., 2015).

Depending on the strain, the FimA protein varies in size from 40.5 to 49 kDa. Based on the amino terminal and the DNA sequences, it is classified into six types: types I–V and Ib (Fujiwara et al., 1993; Hajishengallis et al., 2008). Strains with type IV FimA such as strains W50 and W83, are poorly fimbriated. Strains 381, ATCC 33277, and HG565, on the hand, are abundantly fimbriated type I strains that have significant adhesion to host tissues. It was reported that most of the periodontal patients harbored FimA type II strains followed by type IV (Amano et al., 2004).

Research on the 67-kDa minor fimbria is very much limited. The clonal diversity of minor fimbriae is not well-studied among *P. gingivalis* strains. Similar to FimA protein, distinct minor fimbriae molecules, FimCDE, are found in different strains. A study by Amano et al. (2004) demonstrated that minor fimbriae stimulated the production of interleukin-1α (IL-1α), IL-1β, IL-6, and tumor necrosis factor-α (TNF-α) by macrophages. It is highly postulated that the minor fimbria is a causative factor of alveolar bone resorption in animal models. This study, however, requires further validation as clonal diversity of minor fimbriae is unclear but it is likely to contribute to the progression of human periodontitis.

**FIGURE 3:**
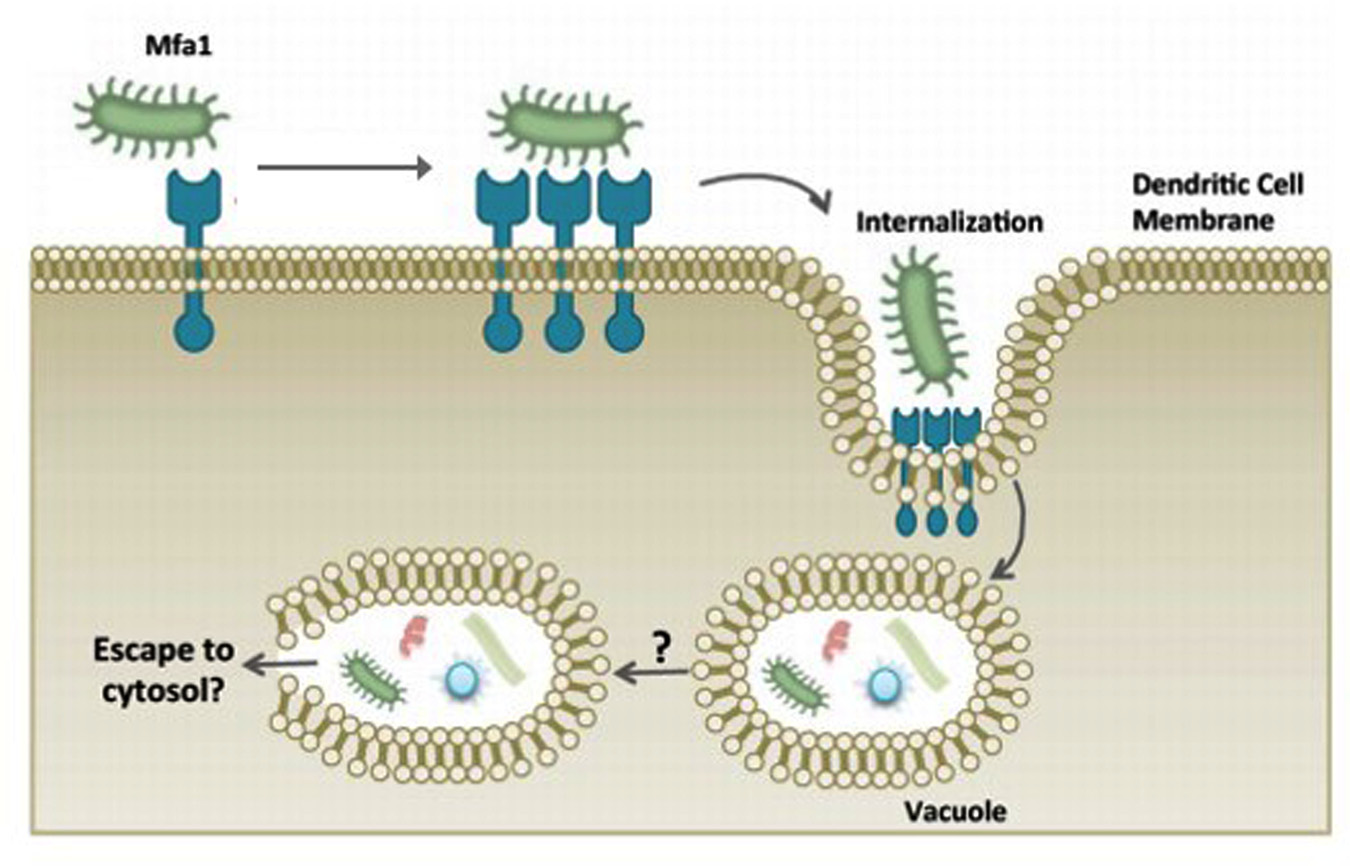
***P. gingivalis* manipulation of dendritic cell entry using minor fimbriae Mfa1 and accessory proteins.** The adhesive properties of fimbriae allows *P. gingivalis* to evade host cells and escape the host immune surveillance. Adapted from Zenobia and Hajishengallis (2015).

Early studies in 1990s on *P. gingivalis* fimbriae deficiency strains revealed the essential roles of fimbriae in the binding as well as invasion of host cells (Lee et al., 1992; Sharma et al., 1993). It also mediates adherence to a wide range of oral substrates and molecules including extracellular matrix proteins, oral epithelial and commensal bacteria such as streptococci and *Actinomyces viscosus* (Amano, 2007). Amano (2010) reported that fimbriae type II is capable of adhering to cellular α_5_β_1_-integrin which enables the bacteria to be easily engulfed by host phagocytes and dendritic cells and causes actin cytoskeleton rearrangement to facilitate internalization. Subsequently, this allows the intracellular bacteria to impair the host cellular function with their virulence factors (Zenobia and Hajishengallis, 2015; **Figure 3**). It was reported that the major fimbriae is involved in the initial invasion of osteoblasts by *P. gingivalis*, but are not essential for the subsequent inhibition of osteoblast differentiation and mineralization (Zhang et al., 2011).

Many reports argue the binding of FimA residues in the fimbriae to the target molecules, but it is reported that minor components of FimCDE critically function as an adhesin (Yoshimura et al., 2009). FimA protein was not essential for the invasive capabilities of *P. gingivalis* outer membrane vesicles. Nevertheless, higher number of protein components associated with vesicles derived from a fimbriated strain are internalized into human gingival fibroblasts in comparison to a fimbriated strain (Mantri et al., 2015). In addition, *P. gingivalis* fimbriae act as an important virulence factor in atherosclerosis progression. It was reported that *P. gingivalis* infection reduced regulatory T cells (Tregs) in atherosclerotic patients. Tregs may play a crucial role in autoimmune response during this process. However, whether *P. gingivalis* infection is associated with Tregs dysregulation during atherosclerosis is still unknown and the prevalence of different *P. gingivalis* FimA genotypes during this process remains unclear (Yang et al., 2014).

Meanwhile, there are various plant-derived compounds used to inhibit activity of *P. gingivalis*. Two of these studies were performed by Murakami et al. (2015) on anti-inflammatory activity of quercetin, resveratrol, and its related compounds, catechin, epicatechin, orcinol, and 4-allylphenol. It was demonstrated that the natural compounds exhibited inhibitory effect on the activity of *P. gingivalis* fimbriae. However, it should be noted that more studies on different strains of *P. gingivalis* are needed to support the aforementioned hypotheses as strain variability may have different pathogenicity.

## Lipopolysaccharide (LPS)

**FIGURE 4:**
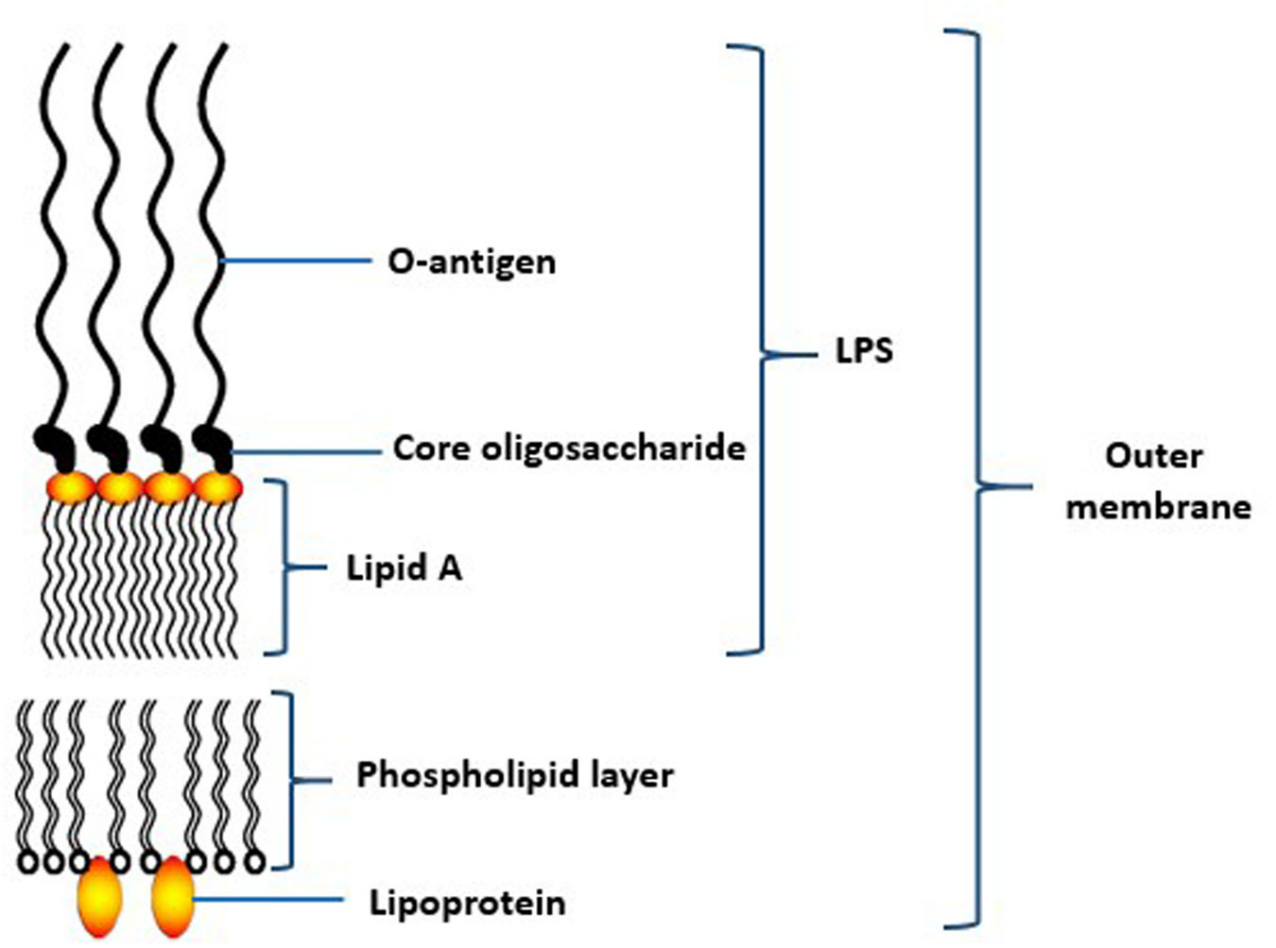
**Schematic structure of lipopolysaccharide (LPS) of the outer membrane of *P. gingivalis*.** Adapted from Ogawa and Yagi (2010).

Lipopolysaccharide (LPS) is a relatively large molecule with at least 10 kDa in size. It constitues an important component of the bacterial outer membrane (Hamada et al., 1994). In general, the bacterial LPS consists of a distal polysaccharide (or O-antigen), a non-repeating “core” oligosaccharide and a hydrophobic domain known as lipid A (or endotoxin) (Ogawa and Yagi, 2010; **Figure 4**). Lipid A, the inner-most component, is the biological active region of LPS that could cause deregulation of the mammalian innate immune system by interacting with both toll-like receptors 2 and 4 (Darveau et al., 2004). It has heterogeneous acylation patterns which change according to microenvironmental conditions and affect the host immune signaling, thereby facilitating bacterial survival in the host (Al-Qutub et al., 2006).

In Gram-negative bacteria, LPS plays critical roles for maintainance of the cellular and structural integrity, as well as a controlling the entry of hydrophobic molecules and toxic chemicals. In fact, the folding and insertion of outer membrane protein could not take place in the absence of LPS (Silhavy et al., 2010). Based on its biological activity, LPS is found to be an important pathogenic factor among many periodontopathic bacteria. A number of investigations have reported the ability of this outer membrane component to activate the host inflammatory responses and disrupt bone remodeling process (Herath et al., 2011; Kato et al., 2014). In clinically healthy periodontium, a normal inflammatory response is a fundamental component of the host defense system with the production of IL-1. IL-1 is believed to play a role in the pathogenesis of periodontal disease through inflammatory reactions through activation of toll-like receptor 4 (Bartold and Narayanan, 2006; Jain and Darveau, 2010).

One possible role of LPS in *P. gingivalis* is to disrupt the innate host surveillance by interfering with distribution of leukocytes in the vicinity of bacterial colonization. In fact, *P. gingivalis* LPS is poorly recognized by innate host defense system compared with the LPS of other Gram-negative species (i.e., *Escherichia coli*) (Liu et al., 2008). Other than that, the ability of gingival epithelial cells to secrete chemokine interleukin 8 (IL-8) was paralyzed, thereby activation of neutrophils, eosinophils, and basophils are affected (Yoshimura et al., 1997; Darveau et al., 1998). This phenomeneon, known as chemokine paralysis, results in resistance to oxidative burst-killing by polymorphonuclear neutrophils. In the absence of an effective innate host immune defense, the number of periodontal bacteria could increase remarkably. The rise of the bacterial population at the gingival area coupled with the failure of host defense system to remove them are consistent with the observed etiology of periodontal disease. Besides inducing pro-inflammatory cytokines synthesis, *P. gingivalis* LPS inhibits osteoblastic differentiation and mineralisation in periodontal ligament stem cells which participate in periodontal tissue regeneration (Kato et al., 2014).

In addition, a recent mice study demonstrated that *P. gingivalis* LPS circulates systemically in more than 50% of periodontal patients and is found to have higher level of matrix metalloproteinase. The low systemic LPS triggers an inflammatory response in the left ventricle through metalloproteinase, thereby causing cardiac dysfunction (DeLeon-Pennell et al., 2013). Apart from that, LPS also stimulates the production of thrombospondin-1, a multifunctional extracellular matrix protein secreted by human monocytic cells. Thrombospondin-1 stimulates macrophage migration and modulates host inflammatory response (Gokyu et al., 2014). The inflammatory responses are mediated by variety of genes. One of them is plasminogen activator inhibitor type I (PAI-1) mRNA-binding protein which was reported to be upregulated in inflammatory gingiva when induced by *P. gingivalis* LPS (Na et al., 2014). Even though PAI-1 plays critical roles in cell migration, it requires further studies to understand the function of plasminogen activator system in cell signaling pathway.

A growing body of evidences shows that several natural compounds exhibit inhibitory effect on LPS. It was reported that alpha-mangostin caused a decrease in IL-6 and IL-8 expression in human gingival fibroblasts (Yiemwattana and Kaomongkolgit, 2015). More recent finding by Ci et al. (2015) demonstrated the inhibitory effect of grape seed proanthocyanidin extracts on *P. gingivalis* LPS while Derradjia et al. (2015) reported the roles of α-tocopherol in countering the damaging effect of LPS by reducing inflammatory cytokines, increasing β-defensins, an antimicrobial peptide, and promoting gingival fibroblast growth and migration. Tormentic acid, on the other hand, was reported to inhibit LPS-induced inflammatory response in human gingival fibroblasts (Jian et al., 2015). As tormentic acid is found to possess anti-inflammatory effect by inhibiting IL-6 and IL-8 production, it can be a possible therapeutic agent for periodontal disease.

In a recent study by Díaz et al. (2015), it was found that differences in LPS profile of *P. gingivalis* clinical isolates affect colony morphology and polymyxin B resistance. Unlike the healthy subjects, isolates from periodontal subjects were resistance to polymyxin B and demonstrated low aggregation ability. This resistance highly correlates with variation in LPS profiles as LPS from healthy subjects lacks of high molecular weight *O*-antigen moieties and anionic polysaccharide (Díaz et al., 2015) whereas *P. gingivalis* isolates from periodontal subjects produce modified lipid-A molecules. However, the role of other LPS components are poorly understood and the participation of other virulence factors in response to antimicrobial compounds were not taken into consideration.

Another important point worth noting is the expression of LPS in various *P. gingivalis* strains, and their roles during various stages of infection still remains controversial. One of the main reasons presumably could be the use of different *P. gingivalis* strains that are inevitably pleiotropic with respect to other pathogenic properties. Hence, the basis for the variation of LPS in different strains has yet to be established.

## Proteases

The ability of most *P. gingivalis* strains to secrete numerous hydrolytic, proteolytic, and lipolytic enzymes along with toxic metabolites, is one of the virulence characteristic that allows these bacteria to thrive in the oral cavity. These enzymes usually come into close proximity with the host cells. While some enzymes are found within the periplasmic space, others are transported from the outer membrane into outer membrane vesicles during growth. One of the type of enzymes, proteases, appear to be strongly implicated in periodontal disease progression. Among these proteases are trypsin-, thiol-, caseinolytic proteinases, and peptidases (Travis et al., 1997; Curtis et al., 2001).

There are generally two distinct families of proteases produced by *P. gingivalis*. One of them is the cysteine proteinase family or also known as “trypsin-like” enzyme and the other one is serine proteinase (Bostanci and Belibasakis, 2012). The “trypsin-like” enzymes cleave polypeptides at the C-terminal after arginine or lysine residue. These proteinases are commonly known as gingipains, namely gingipain R and K, that cleave after arginine and lysine, respectively. They collectively account for 85% of the extracellular proteolytic activity of *P. gingivalis* at the site of infection (de Diego et al., 2014). There are two types of gingipain R, namely RgpA and RgpB, while there is one type of gingipain K, Kgp. Gingipain R degrades extracellular matrix components, including the integrin–fibronectin-binding, cytokine, immunoglobulin and complement factors (Curtis et al., 2001). It is also vital for the processing and maturation of the major fimbriae (FimA) (Kristoffersen et al., 2015). Both gingipain R and K have been isolated from many *P. gingivalis* strains grown under different conditions.

There are strong evidences that indicate *P. gingivalis* proteases involve directly in the colonization of the periodontal pocket, leading to destruction of supporting periodontal tissue (Andrian et al., 2004; Dubin et al., 2013). In addition, the proteases also confer high resistance of the microorganisms to host defense mechanisms. Extensive investigations within the past decade have shown that *P. gingivalis* proteases are involved in the degradation of extracellular matrix proteins such as collagen, activation of the host matrix metalloproteinases, inactivation of plasma proteinase inhibitors, cleavage of cell surface receptors, and deregulation of the inflammatory (Potempa et al., 2000; Imamura et al., 2003). They are also important additive agents on the growth of *T. forsythia* and *A. actinomycetemcomitans* in a mixed-species biofilm with *P. gingivalis* (Bao et al., 2014; Haraguchi et al., 2014).

Gingipain was found to degrade fibrinogen and host heme proteins which contribute to inhibition of blood coagulation and increase bleeding, thereby enhancing the availability of hemin for bacterial growth (Sroka et al., 2001). Hence, it may not be surprising to find high proliferation of *P. gingivalis* within periodontal pockets in which erythrocytes are abundant. Gingipains are also considered important in its capacity to degrade antibacterial peptides, such as neutrophil-derived α-defensins, complement factors, such as C3 and C4, T cell receptors, such as CD4 and CD8 (Hajishengallis et al., 2013; Bao et al., 2014). They also reduce the expression of innate immune receptors CD14, which result in macrophage hyporesponsiveness to bacterial infection. However, whether reduced CD14 expression is linked to periodontitis induced by *P. gingivalis* awaits further clarification. In a recent work by Maekawa et al. (2014), *P. gingivalis* and its gingipains, proactively manipulate host molecules, for instance, by interfering cross-talk between C5a receptor and toll-like receptor signaling to prevent bacterial clearance. *In vitro* studies have demonstrated that gingipains participate in the regulation of inflammatory mediators from various host cells, including IL-1 α, IL-1β, IL-18 (Hamedi et al., 2009), protease-activated receptor (PAR)-2 (Belibasakis et al., 2010), or soluble triggering receptor expressed on myeloid cells (sTREM)-1 (Bostanci et al., 2013). Nevertheless, to this extent, there is no concrete evidence to show simultaneous effect of gingipains on these inflammatory mediators in the progression of periodontitis.

Rhein, an anthraquinone from rhubarb roots, exhibits antibacterial synergistic effect with other polyphenols. It causes a downregulation of two protease genes, rgpA and kgp, which are associated with inactivation of host defense mechanisms and tissue destruction (Azelmat et al., 2015). On the other hand, theaflavins, the main polyphenols in black tea, markedly inhibits the proteinase activities of *P. gingivalis* gingipains in a dose-dependent manner (Kong et al., 2015). A synthetic dual protease inhibitor, termed as KYT-41, was synthesized and found to possess potent antibacterial activity against *P. gingivalis*. This dual inhibitor of Rgp and Kgp proteases exhibit anti-inflammatory activity, thereby is thought to be a promising agent for preventing and treating gingivitis (Kataoka et al., 2014).

## Outer Membrane Proteins

The cell envelope of Gram-negative bacteria such as *Porphyromonas* sp. comprises of two cell membranes, the outer membrane (OM) and the inner membrane (IM). Both layers of membranes have different composition and structure. They are separated by the periplasm containing the peptidoglycan layer. While the IM is a phospholipid bilayer with numerous integral IM proteins (Bos et al., 2007), the OM is an asymmetrical bilayer which consists of phospholipids and lipopolysaccharide in the inner and outer leaflet, respectively. The bacterial cell membrane plays a role as the selective barrier that offers protection and allows the movements of various substances through OM porin proteins (Nikaido, 2003). The OM proteins are generally divided into two categories, the OM lipoproteins that are anchored to the OM by an N-terminal lipid tail, and another is OM integral proteins which contain membrane-spanning regions (Bos and Tommassen, 2004).

The OM is involved in most of the specific recognition processes as it is the most exposed region of a bacterial cell. As relatively few major proteins exist in the OM region, these proteins are expected to be important antigens to the host. The formation and maintenance of periodontal biofilms is postulated to be associated with the interaction among periodontal microflora which is mediated by OM proteins (Bos et al., 2007). There are evidences that T helper cells of aggressive periodontitis patients activated with *P. gingivalis* OM proteins produced higher levels of pro-inflammatory cytokines such as IL-1β and IL-6 in comparison with healthy controls (Gonzales et al., 2014).

Analysis of proteins extracted from the OM using sodium dodecyl sulfate-polyacrylamide gel electrophoresis (SDS-PAGE) revealed the OM consists of a diverse array of proteins ranging in size between 20 and more than 100 kDa. The most abundant OM proteins consistently identified are porins and OmpA-like proteins (Bos et al., 2007). However, until recently, research on major OM proteins in *P. gingivalis* remains scarce due to hydrophobicity nature of the proteins. Several attempts have been made to characterize the OM proteins using different solubilization methods (Yoshimura et al., 2009).

One of the earliest studies is by Yoshimura et al. (1989) is in which a 75-kDa major OM protein that exists as a high molecular weight oligomer was successfully purified. This protein was demonstrated by Watanabe et al. (1992) to stimulate activation of polyclonal B-cell and production of IL-1 from mouse peritoneal macrophages.

On the other hand, Saito et al. (1997) reported that a 40-kDa OM protein from *P. gingivalis* is an important aggregation factor on the cell surface. In this study, the antibody against the protein inhibited the aggregation activity of *P. gingivalis* cells toward *A. viscosus*, which is one of the pioneers to colonize the tooth surfaces. This 40-kDa OM protein is found in many strains of *P. gingivalis*, residing both at the cell surface and in extracellular vesicle. Transcutaneous immunization of mice with 40 kDa OM protein elicited an increase in IgG antibody which inhibited the coaggregation of *P. gingivalis* vesicles (Maeba et al., 2005). In recent years, in animal model studies by Zhang et al. (2009) and Liu et al. (2010), vaccination of mice with 40-kDa OM protein of *P. gingivalis* was demonstrated to elicit a significant reduction of alveolar bone loss.

The 40-kDa OM protein is also a hemin-binding protein (Shibata et al., 2003). Meanwhile, nasal immunization with 40kDa OM protein plus cholera toxin elicits protective immune response against *P. gingivalis* in both young and aged mice besides preventing atherosclerosis (Koizumi et al., 2008; Cai et al., 2013). Together, the results suggest that nasal delivery of OM protein could be a potential vaccine strategy to provide protective immunity to human and hence prevention of periodontitis.

In addition, the OM protein profiles of *P. gingivalis* ATCC 33277 and W83 were compared, and it was found that both strains possess most OM proteins, such as RagA, RagB, and OmpA-like proteins in similar distribution pattern (Imai et al., 2005). These findings, using inmmunoblotting, suggest that these OM proteins are surface-located immunoreactive antigen but cross-reaction with other *P. gingivalis*-related strains has to be taken into consideration.

According to Chen et al. (2011), an OM protein, LptO (PG0027) was found to be essential for the *O*-deacylation of LPS of *P. gingivalis* and such structural formation is postulated to be essential to provide attachment to host cells. It also plays important role in secretion of gingipain to the cell surface. Using blue native PAGE analysis, LptO was found in up to seven different complex sizes and it interacted with another protein complex, PG0026, in secretion of C-terminal domain proteins (Glew et al., 2014; Saiki and Konishi, 2014). Apart from that, a novel OM protein, coined as PG534, was found to play important roles in producing active gingipains (Saiki and Konishi, 2010). The activities of gingipains R (RgpA and RgpB) and gingipain K (Kgp) in *P. gingivalis* with defective PG534 protein were reduced in relative to its wildtype strain.

There are also studies to attenuate the effect *P. gingivalis* OM proteins. For instance, studies on natural plant extracts such as polyphenols of *Myrothamnus flabellifolia* was shown to reduce *P. gingivalis* adhesion and invasion up to about 50% by interacting with bacterial OM proteins. Such anti-adhesive effect is also accompanied by cytoprotective effects which relates to cytokine secretion (Löhr et al., 2011). However, it has to be noted that this plant extract was only tested on OM proteins but not on other virulence components of *P. gingivalis*.

## Conclusion

Taken together, *P. gingivalis* is a major etiological agent in the development of chronic periodontitis. This secondary colonizer is found to express a plethora of virulence factors involved in colonizing the subgingival plaque and modulating the immune responses of the host cells. In order to increase survival into the host, *P. gingivalis* is able to locally invade periondontal tissue, thereby avoiding the immune surveillance while maintaining its viability. Each virulence factor plays important roles to hamper the cell-mediated immune response in host. Nevertheless, in spite of the convincing data presented in this study, it should be cautioned that in an actual *in vivo* situation, the bacteria express a whole subset of virulence factors that may interact with and stimulate host cells in a different way than a single virulence factor. In addition, periodontal disease is rarely the result of single bacteria. Hence, *P. gingivalis* is likely to work with other oral microbiota to thrive in a harsh inflammatory condition in periodontal pocket.

To date, numerous studies have been done to elucidate the mechanism of virulence compounds secreted by *P. gingivalis* and the cellular interaction with the host. Improved understanding of the interaction between periodontal bacteria and host cells at the molecular and cellular level, may ultimately have relevance to the overall well-being of the host. In recent years, the use of natural compounds has gained more attention to attenuate the action of *P. gingivalis*. Plant-derived natural products have been widely explored as the therapeutic roles in regulating interactions between microorganisms. One of the appealing therapeutic feature is bioactive compounds from plants appear to be safe and should not cause toxicity toward human cells. However, a comprehensive toxicity studies on these compounds are still deemed necessary.

With tremendous progress in biomedical studies, we expect to eventually elucidate the possible unique mechanism of *P. gingivalis* and its virulence determinants. Exploration of this field will be of help in the development of effective therapies for controlling bacterial-induced connective tissue destruction. These therapeutics approaches may be important in controlling chronic *P. gingivalis* infections by preventing growth and colonization of *P. gingivalis*.

## Author Contributions

KH analyzed literature and wrote the paper, KS and KC edited and approved the manuscript.

## Conflict of Interest Statement

The authors declare that the research was conducted in the absence of any commercial or financial relationships that could be construed as a potential conflict of interest.
